# Neutrophil activation in systemic capillary leak syndrome (Clarkson disease)

**DOI:** 10.1111/jcmm.14381

**Published:** 2019-06-18

**Authors:** Zhihui Xie, Douglas B. Kuhns, Xuesong Gu, Hasan H. Otu, Towia A. Libermann, John I. Gallin, Samir M. Parikh, Kirk M. Druey

**Affiliations:** ^1^ Lung and Vascular Inflammation Section, Laboratory of Allergic Diseases NIAID/NIH Bethesda Maryland; ^2^ Neutrophil Monitoring Laboratory NCI/NIH Frederick Maryland; ^3^ Genomics, Proteomics, Bioinformatics and Systems Biology Center, Division of Interdisciplinary Medicine and Biotechnology Beth Israel Deaconess Medical Center & Harvard Medical School Boston Massachusetts; ^4^ Department of Electrical and Computer Engineering University of Nebraska Lincoln Nebraska; ^5^ Clinical Pathophysiology Section NIAID/NIH Bethesda Maryland; ^6^ Department of Medicine, Division of Nephrology and Center for Vascular Biology Research Beth Israel Deaconess Medical Center & Harvard Medical School Boston Massachusetts

**Keywords:** endothelial cells, neutrophils, proteomics, vascular leak

## Abstract

Systemic capillary leak syndrome (SCLS; Clarkson disease) is a rare orphan disorder characterized by transient yet recurrent episodes of hypotension and peripheral oedema due to diffuse vascular leakage of fluids and proteins into soft tissues. Humoral mediators, cellular responses and genetic features accounting for the clinical phenotype of SCLS are virtually unknown. Here, we searched for factors altered in acute SCLS plasma relative to matched convalescent samples using multiplexed aptamer‐based proteomic screening. Relative amounts of 612 proteins were changed greater than twofold and 81 proteins were changed at least threefold. Among the most enriched proteins in acute SCLS plasma were neutrophil granule components including bactericidal permeability inducing protein, myeloperoxidase and matrix metalloproteinase 8. Neutrophils isolated from blood of subjects with SCLS or healthy controls responded similarly to routine pro‐inflammatory mediators. However, acute SCLS sera activated neutrophils relative to remission sera. Activated neutrophil supernatants increased permeability of endothelial cells from both controls and SCLS subjects equivalently. Our results suggest systemic neutrophil degranulation during SCLS acute flares, which may contribute to the clinical manifestations of acute vascular leak.

## 1 INTRODUCTION

Systemic capillary leak syndrome (SCLS) is an extremely rare and potentially fatal disorder of unknown aetiology. SCLS is characterized by recurrent, spontaneous attacks of hypovolemic shock and anasarca due to sudden and massive leakage of intravascular fluids and proteins into peripheral tissues. Termed “Clarkson disease” in 1960 for its discoverer, SCLS is diagnosed based on a unique constellation of signs and symptoms including severe hypotension, elevated haematocrit resulting from hemoconcentration and hypoalbuminemia due to leakage from the intravascular space.^1^ Patients are frequently misdiagnosed initially with other conditions such as sepsis and treated with aggressive intravenous fluid resuscitation. Such therapy induces massive “third spacing” of fluids and solutes into peripheral tissues and development of compartment syndromes, which may require fasciotomies. There are no specific treatments for acute flares of SCLS beyond haemodynamic stabilization, nor is there curative therapy.

Specific aetiological factors and pathways contributing the profound vascular barrier breakdown characteristic of acute SCLS flares are unknown. Although more than 80% of SCLS patients have a monoclonal gammopathy of unknown significance, there is no direct or indirect evidence that the monoclonal IgG has any role in disease pathogenesis.^2^ In our studies of more than 65 patients with a confirmed diagnosis of SCLS, we found that several inflammatory cytokines (eg TNFα, CCL2, CXCL10) and mediators of vascular permeability (VEGF, Angpt‐2) were significantly elevated in sera from patients during acute episodes compared to convalescent intervals.^2^, ^3^ Treatment of normal vascular endothelial cells (ECs) with acute but not remission sera from these patients disrupted vascular integrity through mechanisms including internalization of VE‐cadherin and actin stress fibre formation.

In order to broaden our search for mediators unique to SCLS and/or uncover a signature of humoral factors characteristic of acute attacks, we used the slow‐off rate modified aptamer (SOMA)scan platform, an aptamer‐based highly multiplexed proteomic assay capable of detecting more than 1300 protein analytes simultaneously across the whole dynamic range from only a small volume of plasma with high sensitivity and specificity.^4^ The SOMAscan approach uncovered more than 600 proteins, whose relative amounts were increased more than twofold in acute plasma relative to matched baseline plasma from the same patients. Most notably, several neutrophil granule proteins were profoundly elevated in acute SCLS plasma. Acute but not remission sera from SCLS patients activated neutrophils, and supernatants from activated neutrophils induced hyperpermeability of ECs. These results suggest that activated and/or degranulated neutrophils contribute to the pathogenesis of acute SCLS.

## 2 MATERIALS AND METHODS

### 2.1 Subjects

Patients were diagnosed with SCLS according to established criteria, specifically a history of one or more transient episodes of hypotension, elevated haematocrit and hypoalbuminemia.^5^, ^6^ Patients were seen at the Clinical Center of the National Institutes of Health. Written informed consent was obtained from each patient and the study protocol (I‐0184) conformed to the ethical guidelines of the 2008 Declaration of Helsinki, having been approved by the Institutional Review Board of the National Institute of Allergy and Infectious Diseases of NIH. EDTA plasma and sera were collected and stored from both remission and active disease intervals where available. Neutrophils were available only from asymptomatic patients. Anonymized age‐, sex‐ and race‐matched blood from healthy volunteers were obtained from the NIH Blood Bank for neutrophil isolation and used as controls.

### 2.2 Cells and reagents

Human microvascular endothelial cells (HMVECs) and the culture medium EGM2 were purchased from Lonza. Blood outgrowth endothelial cells (BOECs) were generated and cultured as described previously.^7^ Lipopolysaccharide (LPS), cytochalasin B and N‐formyl‐Met‐Leu‐Phe (fMLP) were obtained from Sigma‐Aldrich. Human recombinant VEGFA‐165 and TNFα were from PeproTech. Antibodies anti‐human CD11b/BV786, CD16/APC and CD62L/PE were from Biolegend.

### 2.3 SOMAscan and ELISA

Nine pairs of EDTA‐plasma samples obtained at or near the onset of an acute SCLS crisis and from the same patient during a convalescent interval were analysed using the SOMAscan Assay Kit for human plasma 1.3k V3.2 (SomaLogic, Inc, cat.#900‐00011) according to the standard protocol for EDTA plasma from the manufacturer's and as described previously.^8^ Five pooled human plasma controls and one buffer control were run on the same plate with the test samples for calibration and normalization. Median normalization and calibration of the data were performed according to the standard quality control protocols at SomaLogic. All samples passed the established quality control criteria. SOMAscan data were analysed using Partek Genomics Suite and statistical significance determined using two‐way ANOVA. Proteins enriched in acute *vs* remission plasma at least threefold (*P* < 0.01) were used to generate the Heatmap. Functional enrichment was determined using Ingenuity Pathway Analysis software (Qiagen) using Fisher's Exact with FDR multiple test correction. ELISAs for elastase, myeloperoxidase (MPO), α‐defensin, lactoferrin and MMP‐9 were performed according to the manufacturer's instructions (Cayman Chemical for MPO; R&D Systems for the remainder).

### 2.4 Neutrophil isolation

Neutrophils were isolated from whole blood using Hypaque Ficoll separation. Briefly, 1:1 PBS diluted blood was added onto the top of Ficoll (lymphocyte separation medium, MP Biomedicals), centrifuged at 800x *g* at room temperature for 20 minutes with the brake off. Neutrophils were retrieved from the bottom layer containing RBCs. After RBC lysis using ACK lysing buffer, cell pellets were washed twice in PBS. Neutrophil purity was routinely ~95% based on surface marker expression (CD16^+^, CD3^−^/CD19^−^) as determined by flow cytometry.

### 2.5 Neutrophil activation/degranulation

For analysis of neutrophil activation, freshly isolated neutrophils were incubated with buffer or stimuli in culture medium at 37°C for the indicated time periods, followed by termination of the reaction by addition of ice‐cold PBS. Cells were then stained with Live/Dead violet (ThermoFisher), washed once and stained with anti‐human CD11b/BV785, CD16/APC and CD62L/PE. The samples were then analysed using an LSRII flow cytometer and the data processed using FlowJo software (BD BioSciences). MPO levels were determined by ELISA (Cayman Chemical). Briefly, purified neutrophils were incubated with fMLP (100 nmol/L) for 15 minutes in complete RPMI at 37°C in the presence of cytochalasin B (10 mol/L). Supernatants were collected and analysed by ELISA according to the manufacturer's instructions.

### 2.6 Endothelial permeability measurements

An electric cell‐substrate impedance sensing (ECIS) assay was used to assess endothelial barrier function. Electrical resistance was measured across endothelial monolayers at 4,000 Hz using the ECIS Zθ apparatus (Applied BioPhysics) as described previously.^2^ Briefly, ECs were serum starved in endothelial basal medium (EBM2, Lonza) plus 0.2% BSA for 5 hours followed by addition of test reagents. Resistance was recorded over a period of 20 hours. Each condition was measured in duplicate in a single experiment and averaged. Absolute resistance values were normalized by subtracting the resistance at time zero (pre‐treatment); the maximal change in resistance was calculated as percentage change over time zero.

### 2.7 Statistical analysis

ELISA and flow cytometric data were analysed with GraphPad Prism 7 software package. Data were analysed by two‐way ANOVA with multiple corrections testing, and non‐parametric tests were used for flow data analysis (Mann‐Whitney test for analysis of two groups; Kruskal‐Wallis for analysis of multiple groups). *P* < 0.05 were considered significant.

## 3 RESULTS

### 3.1 Proteomic profile of acute SCLS plasma

**Figure 1:**
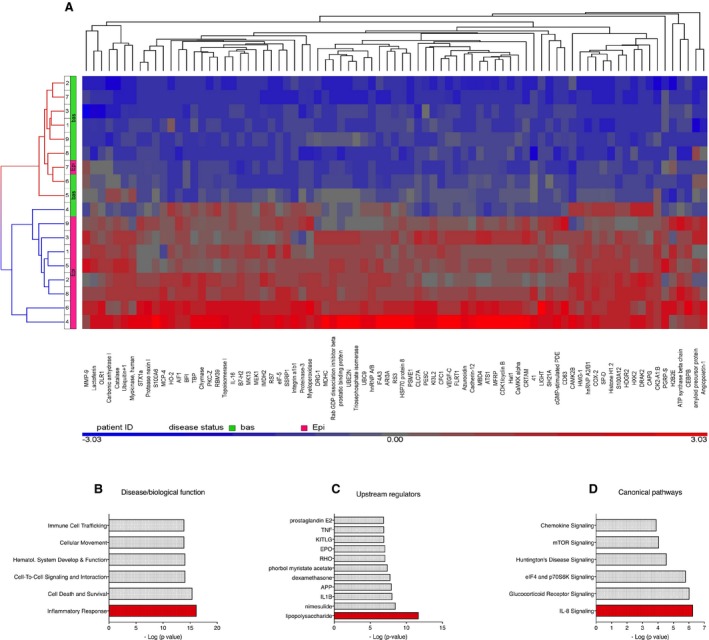
Proteomic profiling of systemic capillary leak syndrome (SCLS) plasma. Nine pairs of matched acute‐remission plasma from SCLS patients were analysed using SOMAscan. (A), Heatmap representation of relative protein amounts. (B‐D), Disease associations determined by Ingenuity pathway analysis and represented by *P*‐values (Fisher's exact test)

Although the clinical symptoms of acute SCLS crises, including hemodynamic collapse and anasarca, are quite dramatic, patients are typically asymptomatic in between episodes. Although SCLS flares may occur in the absence of obvious triggers, recent survey studies suggest that infections (typically upper respiratory) precede SCLS attacks in 35%‐50% of adult patients.^1^, ^9^ These findings suggest that infection‐related inflammatory mediators may induce vascular leakage in some patients. Indeed, in limited prior screening of acute SCLS sera, we found elevated levels of pro‐inflammatory mediators including TNFα, CXCL10, CCL2 and IL‐8.^2^, ^3^ To more fully probe the SCLS proteome, we performed SOMAscan profiling of 1305 proteins in nine matched plasma samples obtained by venipuncture during acute or convalescent intervals. The characteristics of these patients are described in Table 1. This assay identified 612 proteins, whose relative amounts were changed at least twofold in episodic plasma relative to baseline (*P* < 0.05) (full list in Table S1), and all but three of them were increased. Eighty‐one proteins were increased at least threefold in episodic plasma (*P* < 0.001) (Figure 1A). With one exception, the protein profile of episodic and baseline samples clustered with one another. Consistent with previous studies of our entire SCLS cohort, several cytokines and vascular permeability mediators including CCL2, CXCL10, IL‐12, IL‐1β, TNFα, adrenomedullin, Angpt‐2 and VEGFA were significantly elevated in episodic plasma compared to remission plasma (Table S2).^2^, ^3^, ^10^ We also discovered enrichment of several proteins not previously associated with SCLS including surfactant protein D S(P‐D), capping actin protein (CAPG), chymase, allograft inflammatory factor (AIF1), the TNFα superfamily member LIGHT and the stressorin IL‐16 (Table 2). We analysed functional enrichment using Ingenuity pathway analysis. In the category of “Disease & Biological Function”, “Inflammatory response” was the statistically most significant category (*P* = 10^−15^) (Figure 1B); “Upstream Regulators” enriched in acute SCLS samples included several pro‐inflammatory mediators such as LPS, IL‐1β and TNFα (all *P* < 10^−6^) (Figure 1C). Finally, the top “Canonical Pathways” implicated in acute SCLS were IL‐8 and glucocorticoid signalling (*P* = 10^−6^) (Figure 1D).

**Table 1:** Characteristics of SCLS patients

Sample #	Sex	Age at diagnosis	#Episodes	Complications	Current treatment
1	M	63	>10	None	IVIG
2	F	40	4	CS, neuropathy	IVIG
3	M	48	>20	CS, neuropathy, thrombosis, GI infarct	IVIG
4	M	48	2	None	IVIG
5	M	46	>10	None	IVIG
6	M	43	11	CS	IVIG
7	M	68	>10	CS	IVIG
8	F	48	2 (+chronic)	Diarrhoea, vomiting, wasting	None (deceased)
9	F	37	2	Cardiac tamponade	IVIG

Abbreviation: CS, compartment syndrome; SCLS, systemic capillary leak syndrome

**Table 2:** Novel systemic capillary leak syndrome‐associated proteins

Protein	*P*‐value	Fold change (Epi/Bas)	Functional annotation (Ref)
Surfactant protein D (SP‐D)	0.003019	13.4	Immunomodulatory (22)
Capping actin protein (CAPG)	0.001301	6.8	Gelsolin‐related (actin binding protein); inflammation (23)
Chymase	0.000384	6.56	Mast cell‐derived (24)
Allograft inflammatory factor 1	0.000172	4.36	Actin‐bindin protein; pro‐inflammatory (25)
LIGHT	0.001306	3.99	TNFα superfamily (26)
IL‐16	0.000697	3.05	Stressorin (27)

### 3.2 Neutrophil activation signature in SCLS disease flares

**Figure 2:**
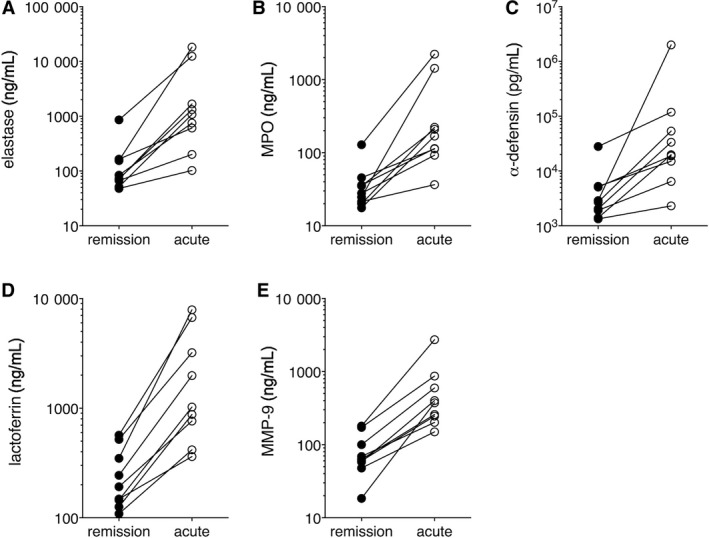
Levels of neutrophil granule constituents in systemic capillary leak syndrome plasma. (A‐E), Levels of the indicated neutrophil granule components were determined in matched acute‐remission plasma samples used in SOMAscan screening by ELISA

Among the proteins that were increased the most in acute SCLS plasma were neutrophil granule components bactericidal permeability increasing protein (BPI) (19.5‐fold), matrix metalloproteinase 8 (MMP8, 7.9‐fold) and MMP9 (4.5‐fold) (Table 3). As noted above functionally enriched pathways in acute samples included signalling involving IL‐8, a well‐known neutrophil chemoattractant, and LPS, which also activates neutrophils.^11^ Given these findings, we hypothesized that neutrophil degranulation and activation may contribute to SCLS flares. To confirm the presence of systemic neutrophil degranulation in SCLS, we measured levels of granule components in the same plasma samples used for SOMAscan screening by quantitative ELISA. Contents of primary granules including elastase, MPO and α‐defensin, secondary granules (lactoferrin) and tertiary granules (MMP‐9) were significantly increased in episodic SCLS plasma compared to baseline in all patients (Figure 2A‐E), confirming the SOMAscan‐derived data. Although total neutrophil counts were not available from the date the acute plasma were drawn, absolute neutrophilia is not a consistent feature of acute SCLS attacks in our cohort as a whole although total white blood cell counts may be elevated due to infection and/or hemoconcentration. Indeed, protein extravasation and reduced blood volume may confound interpretation of plasma protein quantities during an SCLS flare. To evaluate the contribution of such factors, we normalized raw SOMAScan values for (MPO, molecular weight 150 kDa) by those for proteins of comparable size, whose relative values were unchanged in matched acute and remission samples. MPO corrected for either NrCAM (molecular weight [mw] 140 kDa) or MET (mw 145 kDa) values were similar to uncorrected values (Figure S1). Thus, we conclude that hemoconcentration alone is unlikely to account for the increased MPO in acute SCLS plasma relative to convalescence. Instead, our findings suggested that widespread neutrophil degranulation occurs in circulation during SCLS crises.

**Table 3:** Neutrophil granule‐associated proteins in acute systemic capillary leak syndrome sera

Protein	p‐value	Fold change (Epi/Bas)	Granules
Bactericidal permeability increasing protein (BPI)	1.65E‐05	19.52	Azurophilic/primary
Myeloperoxidase (MPO)	0.0009012	3.07	Azurophilic/primary
Cathepsin G	0.0011997	2.80	Azurophilic/primary
Matrix Metalloproteinase 8 (MMP8)	0.0190632	7.93	Specific/secondary
Gelatinase (MMP9)	0.0004257	4.54	Tertiary
MMP1	0.0023533	2.62	Tertiary
MMP13	0.0057115	1.66	Tertiary

Significantly enriched neutrophil‐associated proteins identified by SOMAscan are listed in the table along with their granule locations.

### 3.3 Intrinsic neutrophil function in SCLS

**Figure 3:**
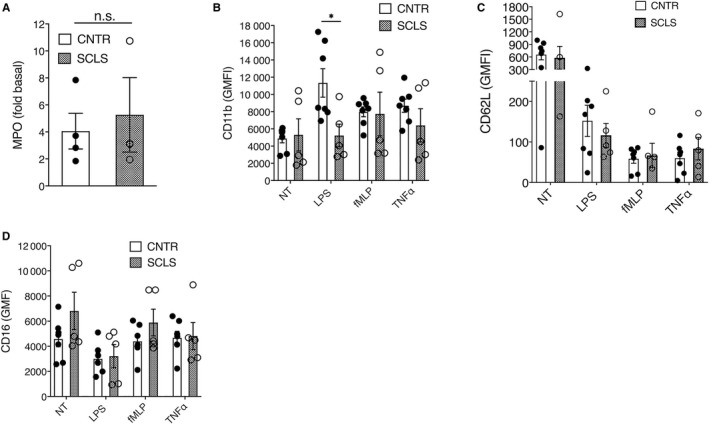
Responses of neutrophils from systemic capillary leak syndrome (SCLS) subjects to pro‐inflammatory mediators. Neutrophils were isolated from whole blood and incubated left untreated (no treatment, NT) or treated with fMLP for 15 minutes in the presence of cytochalasin B. (A), myeloperoxidase (MPO) released was measured by ELISA, and the data are expressed as the fold change in MPO compared to unstimulated cells (mean ± SEM). (B‐D), Purified neutrophils were not treated (NT) or exposed to Lipopolysaccharide (LPS), fMLP or TNFα for one hour. Cells were immunostained with antibodies against the indicated markers and surface expression analysed by flow cytometry. Data are expressed as geometric mean florescence intensity (GMFI) (mean ± SEM); *P < 0.05, Mann‐Whitney *u* test

We next determined whether neutrophils from SCLS patients are intrinsically hyper‐responsive to inflammatory stimuli, which could account for the increased granule components in acute plasma. We first measured neutrophil oxidative burst using a dihydrorhodamine assay. In response to phorbol myristate acetate stimulation, neutrophils from SCLS subjects yielded an average stimulation index of 187.7 ± 2.97, well within the normal range of the testing clinical laboratory ([115‐291], n = 3), indicating that the neutrophil oxidative burst is intact in SCLS. We next measured degranulation by quantifying MPO secretion. Amounts of MPO released following fMLP stimulation of neutrophils from SCLS subjects and healthy controls were indistinguishable from one another (Figure 3A). We also assessed neutrophil activation by quantifying surface marker expression using flow cytometry. Activated neutrophils upregulate CD11b while shedding other membrane‐associated proteins including CD62L (L‐selectin) and CD16. Baseline expression of numerous surface markers was comparable in untouched neutrophils from whole blood of SCLS patients and controls as assessed by flow cytometry, including granulocyte activation markers 31D8, CD63 and CD62L, β2‐integrins and Fc receptors (Figure S2). Likewise, stimulation of purified neutrophils with several proinflammatory mediators including fMLP, TNFα and LPS induced a comparable pattern of activation marker expression in neutrophils isolated from peripheral blood of healthy donors and SCLS subjects during disease‐free intervals (Figure 3B‐D). These results indicate that neutrophils from SCLS subjects retain the capacity to degranulate and release reactive oxygen species (ROS) in response to pro‐inflammatory stimuli.

### 3.4 Acute SCLS sera induce neutrophil activation

**Figure 4:**
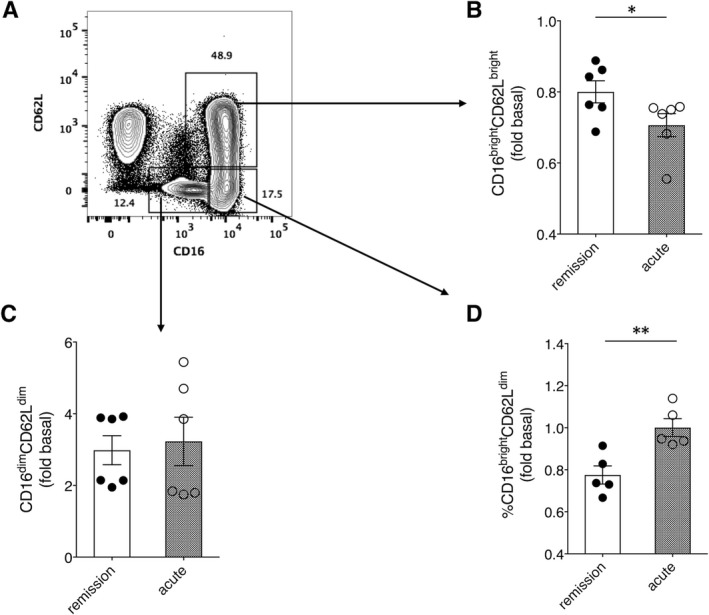
Acute systemic capillary leak syndrome (SCLS) sera elicit neutrophil activation. (A‐C), Purified neutrophils from healthy donors were left untreated or incubated with acute or remission SCLS sera for one hour followed by immunostaining with antibodies against the indicated surface markers and analysis by flow cytometry. Results are expressed as the fold change in the percentage of cells positive for the indicated markers relative to unstimulated cells; *P = 0.04, **P = 0.002, Mann‐Whitney *u* test

A subpopulation of highly activated neutrophils (CD62L^dim^CD16^bright^) is recruited to the circulation from bone marrow in response to acute systemic inflammation associated with sepsis and acute trauma, among others.^12^, ^13^ Given the link between SCLS episodes and infection, and the presence of pro‐inflammatory cytokines in acute SCLS sera, we hypothesized that SCLS sera and/or plasma activate neutrophils. To investigate this, we analysed surface marker expression on purified neutrophils from healthy donors incubated with various sera by flow cytometry, as the availability of plasma was limited. Treatment of neutrophils with acute SCLS sera significantly increased the activated (CD16^bright^/CD62L^dim^) subpopulation, with a concomitant decrease in the CD16^bright^/CD62L^bright^ (quiescent) subset, compared to cells treated with matched remission sera. (Figure 4A‐D). This result suggests that SCLS serum components constrained to acute disease flares activate neutrophils, which in turn could account for the increase in granule components in acute SCLS plasma relative to that from asymptomatic individuals.

### 3.5 Neutrophil degranulation and vascular barrier integrity

**Figure 5:**
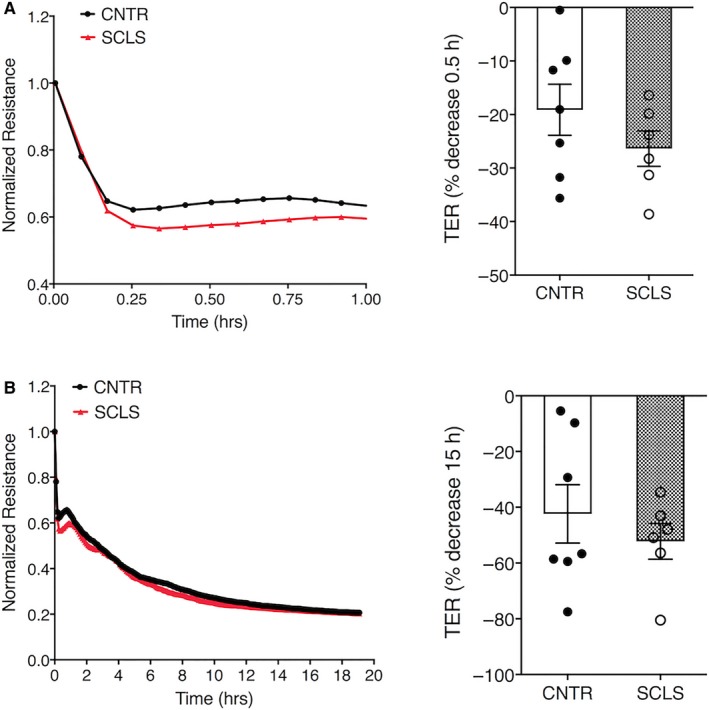
Activated neutrophil supernatants enhance endothelial permeability. (A‐B), Transendothelial resistance (TER) across Blood outgrowth endothelial cells from healthy subjects or systemic capillary leak syndrome (SCLS) patients was measured after application of fMLP‐stimulated neutrophil supernatants for 30 minutes (A) or 15 hours (B). TER over time in a representative experiment is shown on the left; bar graphs on right show the percentage decrease in baseline resistance at the indicated time points (mean ± SEM)

We tested the effects of neutrophil granule contents on endothelial barrier function. First, we stimulated neutrophils isolated from healthy donors with medium alone or fMLP. We confirmed neutrophil degranulation by MPO assay (Figure S3A). We then treated ECs with supernatants from the activated neutrophils and measured transendothelial electrical resistance (TER) across monolayers in real time. fMLP‐stimulated neutrophil supernatants from two separate donors led to decreased TER (ie increased permeability) of HMVECs, while the resistance of cells exposed to medium alone remained relatively constant over a period of 10 hours (Figure S3B). We next determined the effects of degranulated neutrophil supernatants on ECs isolated and expanded from peripheral blood of SCLS patients or healthy controls BOECs.^7^ The activated neutrophil supernatants reduced TER in a pattern similar to commercially supplied HMVECs (Figure 5A,B). However, the maximal decrease in resistance in BOECs from SCLS subjects and controls was equivalent at both early (30 minutes) and later (15 hours) time points. Thus, although neutrophil granule constituents exert permeability‐inducing effects on ECs, our data do not suggest hypersensitivity of SCLS ECs to this stimulus.

## 4 DISCUSSION

From our proteomic screening study of SCLS plasma, we discovered that neutrophils are activated during SCLS disease flares. Neutrophil granule contents (primary, secondary and tertiary granules), including MPO, BPI, Cathepsin G, gelatinase and collagenases, were increased in acute plasma relative to baseline, indicating systemic neutrophil degranulation. Hemoconcentration and protein extravasation are confounding factors for the interpretation of changes in levels of plasma proteins during an SCLS flare. The degree of hemoconcentration in a typical SCLS episode is 50%‐80%, as indicated by the mean change in hematocrit. In comparison, levels of (BPI, mw 25 kDa), the top‐ranked protein from the SOMAScan screen, rose nearly 2000% in acute plasma relative to matched convalescent plasma. As early as 1977, tracking studies of proteins of varying molecular weights in SCLS sera during flares determined that although albumin (mw 62 kDa) levels decreased by 30%‐50% during an episode, C1 (mw 200 KDa) decreased only 10% and IgM (mw 900 kDa) levels rose in direct proportion to the increase in hematocrit.^14^ Thus, proteins up to 900 kDa may extravasate during episodes of SCLS. SOMA screening revealed that MPO (mw 150 kDa) levels increased more than threefold in acute sera relative to baseline, whereas several unrelated proteins of similar size were not elevated in the same samples (eg CHRDL [150 kDa], 1.01‐fold change in basal *vs* episodic sera; NrCAM [140 kDa], −1.00‐fold; MET [145 kDa], 1.003‐fold). In addition, SOMAScan underestimated the increases in MPO, as quantitative ELISA revealed that absolute MPO levels actually increased nearly 10‐fold in episodic *vs* remission plasma (Figure 2B). Thus, we conclude that a) hemoconcentration alone could not account for elevations in circulating concentration of plasma proteins; b) changes in plasma protein levels are not solely based extravasation due to size.

The impetus for neutrophil activation is still unknown. Because many patients have infections preceding flares and most have elevated serum levels of pro‐inflammatory cytokines at the onset of an attack, the factors inciting neutrophil activation may not be unique to SCLS. Neutrophil degranulation occurs in other diseases associated with systemic inflammation including sepsis. Notably, however, mean plasma levels of azurophilic granule components such as MPO in acute SCLS were nearly ninefold higher than those in septic plasma (514 *vs* 60 ng/mL).^15^ Similarly, although elevated levels of tertiary granule constituents including MMP9 are observed in sepsis and other systemic diseases with prominent vascular abnormalities such as Kawasaki syndrome, levels of MMP9 are twofold to fivefold higher in SCLS.^16^

Our results thus far suggest that neutrophils from SCLS patients are not predisposed to degranulate or become activated in response to routine inflammatory mediators. This was somewhat unexpected since we discovered distinct transcriptomes and responses of SCLS ECs and peripheral blood mononuclear cells compared to healthy controls.^7^, ^10^ For example, monocytes and ECs from SCLS patients produced more pro‐inflammatory mediators—including adrenomedullin (ADM) and CXCL10—in response to treatment with SCLS‐associated cytokines (IFNγ) compared to controls.^3^, ^10^ It is possible that SCLS neutrophils may hyper‐respond to a constellation of SCLS‐associated factors that cannot be fully recapitulated in vitro; limited sample availability precludes us from formally testing this hypothesis at the present time through studies of matched patient neutrophils and acute sera.

Clinical manifestations of acute SCLS including hypovolemic shock and anasarca most likely result from disruption of the endothelial barrier of the soft tissue microvasculature.^2^ The current study suggests that neutrophil‐derived mediators contribute to endothelial dysfunction in SCLS. MPO is a phagocytic peroxidase enzyme whose major product is hypochlorous acid, a potent microbicidal agent, generated from hydrogen peroxide (H_2_O_2_), which plays a key role in host responses.^17^ MPO and ROS including H_2_O_2_ have been shown to increase permeability of HMVEC monolayers in vitro.^18^ In addition, MPO has the capacity to sequester nitric oxide (NO), resulting in decreased bioavailability. NO promotes relaxation of vascular smooth muscle and endothelial contraction and has been implicated as a therapeutic target for SCLS‐associated hypotension.^19^ Rodent studies show that MPO localizes at the vascular endothelium and extracellular matrix under inflammatory stress and affects vascular smooth muscle and endothelial functions.^20^ ROS may also induce EC apoptosis; however, we did not observe apoptosis of HMVECs incubated with acute or convalescent SCLS sera.^2^

Another key finding is the increased activation of neutrophils induced by acute but not basal sera, as indicated by the increased percentage of the cells shedding CD62L. A CD16^bright^/CD62L^dim^ neutrophil subset appears to be quite distinct from other neutrophil subpopulations in vivo; published work has suggested that such cells may be recruited from bone marrow to circulation in response to acute inflammation.^13^ This subset is less adherent to ECs and may play an immunosuppressive function as it preferentially migrates to splenic white pulp and inhibits T cell proliferation.^12^, ^21^ Whether this neutrophil subpopulation has a distinct function in SCLS or simply reflects de novo activation requires further study.

**Figure 6:**
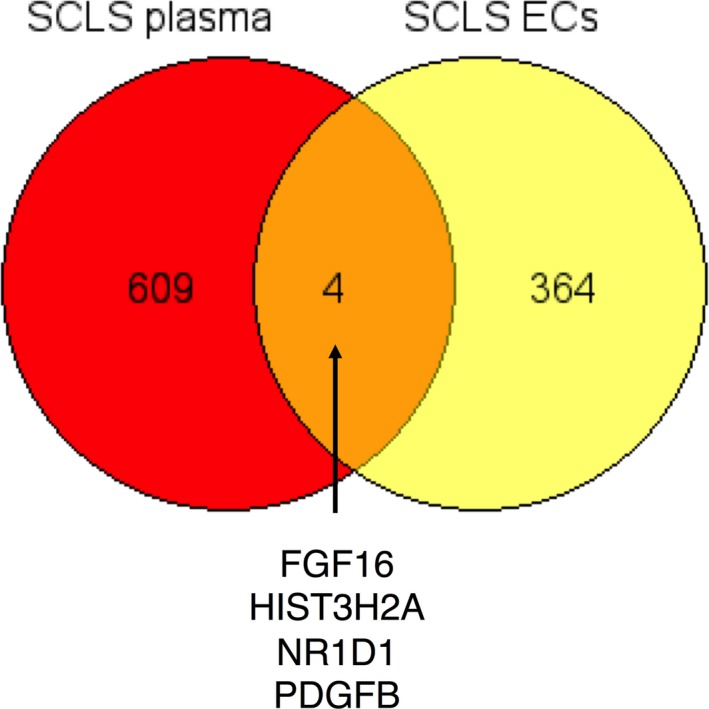
SOMAscan and other discovery‐driven approaches to unraveling systemic capillary leak syndrome (SCLS) disease mechanisms. Analysis of shared genes encoding proteins increased greater than twofold in acute *vs* remission SCLS plasma and transcripts significantly enriched >log2‐fold in SCLS Blood‐outgrowth endothelial cells *vs* controls using GeneVenn. Shared genes listed at bottom

In summary, through multiplexed proteomic screening of plasma, we have discerned a panoply of proteins enriched in plasma during SCLS flares that suggests widespread neutrophil activation/degranulation. Our results demonstrate the utility of SOMAscan as a discovery tool for disease mechanisms. A comparison of genes encoding proteins elevated in acute SCLS plasma with transcripts overexpressed SCLS ECs compared to controls (unpublished RNA‐Seq data) revealed a possible cellular source for several SCLS‐enriched proteins (Figure 6). As but one example, platelet‐derived growth factor B, which is secreted by ECs, stimulates proliferation and activation of pericytes, which are smooth muscle‐like cells surrounding microvessels. Pericyte coverage is required for maintenance of microvascular endothelial barrier function in acute inflammation.^22^ We believe that these and other discovery‐driven approaches will eventually lead to advances in classification of SCLS and development of biomarkers for treatment efficacy.

## CONFLICT OF INTERESTS

The authors declare no conflicts of interest.

## AUTHORS CONTRIBUTIONS

XZ, TAL, DBK, XG, HHO performed experiments and analysed data, and edited the paper. XZ, JIG, SMP and KMD designed and oversaw the project, and wrote and edited the paper.

## DATA AVAILABILITY STATEMENT

The data that support the findings of this study are available from the corresponding author upon reasonable request.
