# Supplementary figures and images for: Neutrophil activation in systemic capillary leak syndrome (Clarkson disease)
Source: J Cell Mol Med. 2019 Jun 18;23(8):5119–27. doi: 10.1111/jcmm.14381 (PMC6653644; doi:10.1111/jcmm.14381)

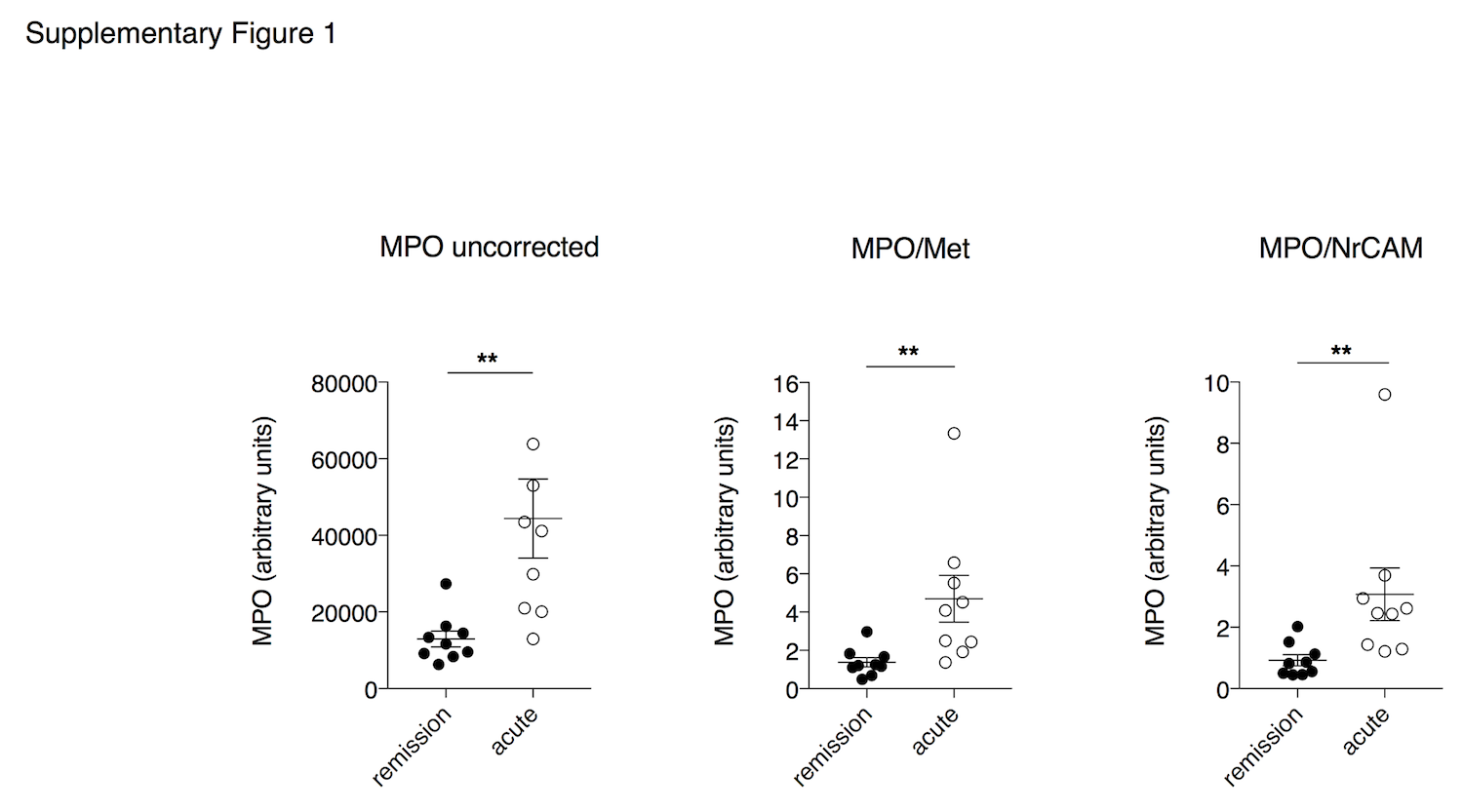

Supplement: Supplementary file 1 [file JCMM-23-5119-s001.tiff]

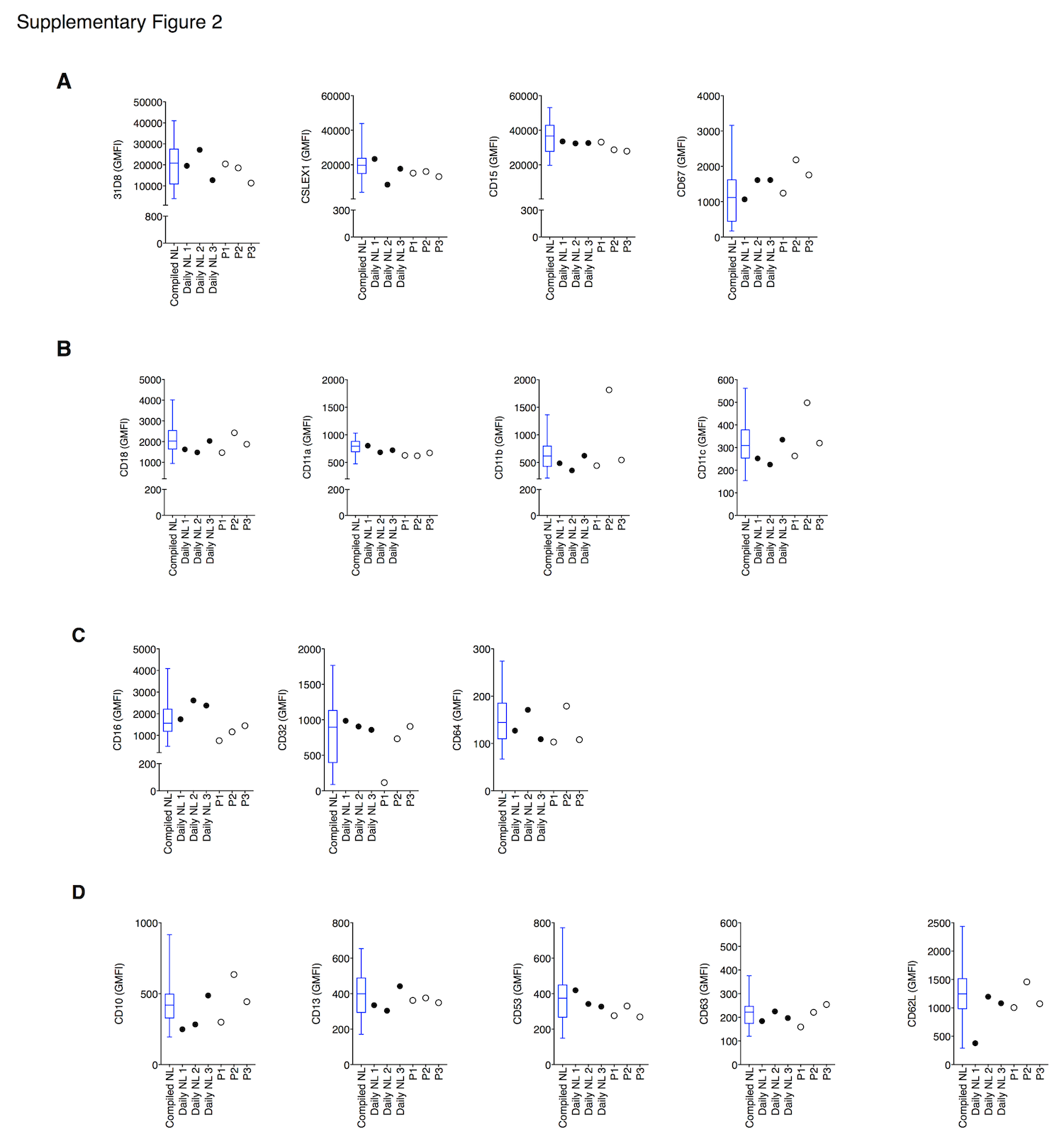

Supplement: Supplementary file 2 [file JCMM-23-5119-s002.tiff]

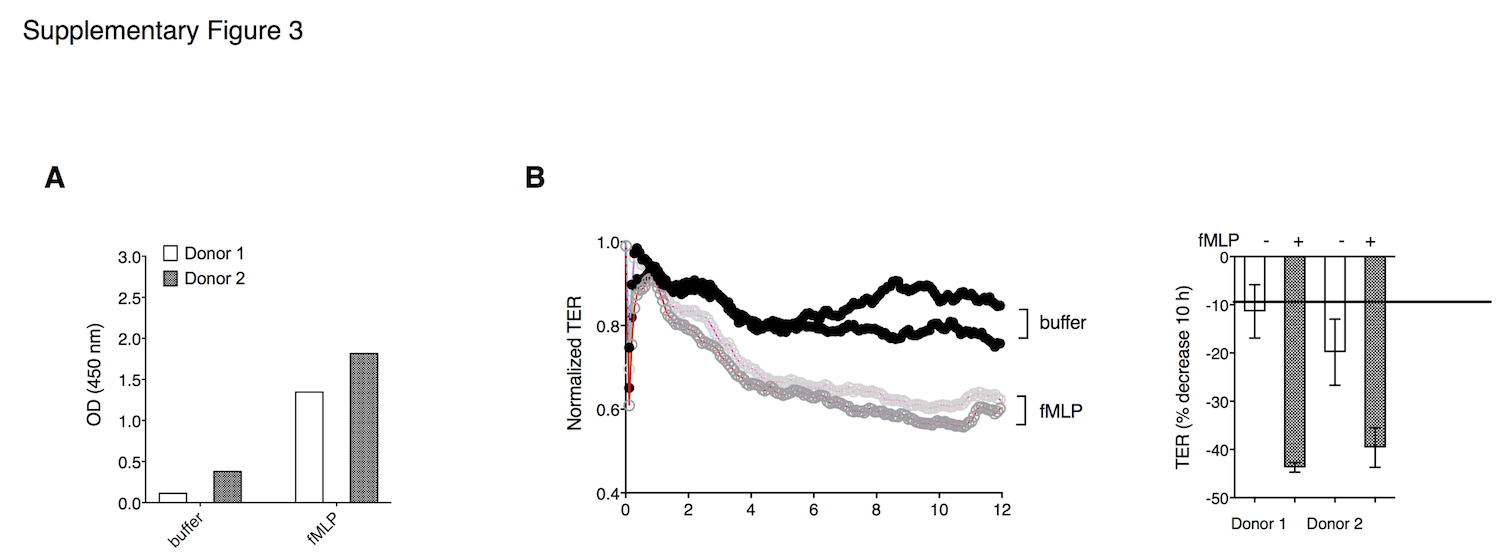

Supplement: Supplementary file 3 [file JCMM-23-5119-s003.tiff]
