# Supplementary material for: Neutrophil activation in systemic capillary leak syndrome (Clarkson disease)
Source: J Cell Mol Med. 2019 Jun 18;23(8):5119–27. doi: 10.1111/jcmm.14381 (PMC6653644; doi:10.1111/jcmm.14381)
Supplement: Supplementary file 5 [file JCMM-23-5119-s005.docx]

**Table 1 Characteristics of SCLS patients**

| **Sample #** | **Sex** | **Age at Diagnosis** | **# Episodes** | **Complications** | **Current Treatment** |
| --- | --- | --- | --- | --- | --- |
| 1 | M | 63 | >10 | None | IVIG |
| 2 | F | 40 | 4 | CS*, neuropathy | IVIG |
| 3 | M | 48 | >20 | CS, neuropathy, thrombosis, GI infarct | IVIG |
| 4 | M | 48 | 2 | None | IVIG |
| 5 | M | 46 | >10 | None | IVIG |
| 6 | M | 43 | 11 | CS* | IVIG |
| 7 | M | 68 | >10 | CS* | IVIG |
| 8 | F | 48 | 2 (+chronic) | Diarrhea, vomiting, wasting | None (deceased) |
| 9 | F | 37 | 2 | Cardiac tamponade | IVIG |

*CS= compartment sydrome

**Table 2 Novel SCLS-associated proteins**

| **Protein** | **p-value** | **Fold Change (Epi/Bas)** | **Functional Annotation (Ref)** |
| --- | --- | --- | --- |
| Surfactant protein D (SP-D) | 0.003019 | 13.4 | Immunomodulatory (22) |
| Capping actin protein (CAPG) | 0.001301 | 6.8 | Gelsolin-related (actin binding protein); inflammation (23) |
| Chymase | 0.000384 | 6.56 | Mast cell-derived (24) |
| Allograft inflammatory factor 1(ALF1) | 0.000172 | 4.36 | Actin-binding protein; proinflammatory (25) |
| LIGHT | 0.001306 | 3.99 | TNFα superfamily (26) |
| IL-16 | 0.000697 | 3.05 | Stressorin (27) |

**Table 3 Neutrophil activation signature in SCLS**

| **Protein** | **p-value** | **Fold Change (Epi/Bas)** | **Granules** |
| --- | --- | --- | --- |
| Bactericidal permeability increasing protein (BPI) | 1.65E-05 | 19.52 | Azurophilic/primary |
| Myeloperoxidase (MPO) | 0.0009012 | 3.07 | Azurophilic/primary |
| Cathepsin G | 0.0011997 | 2.80 | Azurophilic/primary |
|  |  |  |  |
| Matrix Metalloproteinase 8 (MMP8) | 0.0190632 | 7.93 | Specific/secondary |
|  |  |  |  |
| Gelatinase (MMP9) | 0.0004257 | 4.54 | Tertiary |
| MMP1 | 0.0023533 | 2.62 | Tertiary |
| MMP13 | 0.0057115 | 1.66 | Tertiary |

**Supplementary Table 1 Known SCLS-associated mediators**

| **Full name** | **Abbreviation** | **p-value** | **Fold Change (Epi/Bas)** |
| --- | --- | --- | --- |
| C-C motif chemokine 2 | CCL2 | 0.00089842 | 2.42151 |
| Angiopoietin-2 | Angpt-2 | 0.00111697 | 1.86229 |
| Interleukin-1 beta | IL-1b | 0.00190990 | 2.04149 |
| Vascular endothelial growth factor A | VEGF-A | 0.00272506 | 1.51168 |
| Tumor necrosis factor | TNFα | 0.00470640 | 2.08592 |
| Adrenomedullin | ADM | 0.00820154 | 1.62888 |
| Interleukin-12 | IL-12 | 0.00937690 | 2.15551 |
| C-X-C motif chemokine 10 | CXCL10 | 0.03088590 | 2.26489 |
| Interferon gamma | IFN-γ | 0.03142640 | 1.60852 |
